# Muscle function and functional performance after pulmonary rehabilitation in patients with chronic obstructive pulmonary disease: a prospective observational study

**DOI:** 10.1038/s41598-022-20746-y

**Published:** 2022-09-30

**Authors:** Simone Pancera, Luca N. C. Bianchi, Roberto Porta, Jorge H. Villafañe, Riccardo Buraschi, Nicola F. Lopomo

**Affiliations:** 1 IRCCS Fondazione Don Carlo Gnocchi, via Capecelatro 66, 20148 Milan, Italy; 2 Department of Information Engineering, University of Brescia, Brescia, Italy

**Keywords:** Health care, Rehabilitation, Diseases, Chronic obstructive pulmonary disease

## Abstract

This study aimed to measure changes in different properties of skeletal muscles and evaluate their contribution and relationship to changes in functional performance after pulmonary rehabilitation (PR) in patients with chronic obstructive pulmonary disease (COPD). COPD outpatients attending 5 weeks of conventional PR were recruited. Functional performance [5-repetitions sit-to-stand (5STS), and 4-m gait speed (4mGS)], and muscle function (maximal isometric strength, power, force control, and relative concentric and eccentric activation during 5STS) were assessed after PR and 3 months of follow-up. Twenty patients (71 years; 52% of predicted FEV_1_) completed the study. 4mGS and relative concentric activation during 5STS decreased respectively by 7.7% and 26% between the beginning of PR and follow-up. Quadriceps strength, power, and force control improved by 10.4%, 27.3%, and 15.2%, respectively, from the beginning of PR to follow-up the relative eccentric activation during 5STS explained 31% of the variance in 4mGS changes. In conclusion, functional performance appeared to decline after conventional PR, whereas several properties of skeletal muscles were maintained at follow-up in COPD outpatients. Of note, eccentric contractions might play a role in the improvement of functional performance. Therefore, future studies with interventional design should include eccentric training in PR programs during clinical COPD practice.

## Introduction

The dysfunction of peripheral skeletal muscles is prevalent in individuals diagnosed with chronic obstructive pulmonary disease (COPD) and represents a relevant contributor to impairment and disability in these people^1,2^. Weakness of skeletal muscles and loss of fat-free mass are common findings involving 38% and 50% of patients with severe COPD, respectively, and resulting in decreased functional and exercise capacity^3,4^.

Despite its prognostic value, the assessment of muscle function in COPD is not entirely implemented in the clinical setting, or is limited to functional outcome measures (e.g., 5-repetitions sit-to-stand (5STS), and 4-m gait speed (4mGS) tests)^5^. These tests, even if suitable for the outpatient setting, do not provide sufficient quantitative data to comprehensively describe different aspects of skeletal muscle function^6^. In contrast, specific strength measurements (e.g., isokinetic test) might be more informative about skeletal muscle function in COPD but are usually limited to the evaluation of maximal muscle strength or endurance^7^.

In order to provide individualized exercise prescriptions to patients with COPD, several muscle properties that may contribute to skeletal muscle dysfunction need to be assessed^8^. For example, the modality (i.e., isometric, concentric, and eccentric), velocity (i.e., muscle power and rate of force development (RFD)), and fluctuation (i.e., force steadiness) of muscle contraction produce different effects on skeletal muscle function in older healthy adults^9^. Previous studies showed that older subjects had lower maximal strength, slower force production, and more force variability compared with young subjects^9,10^. Moreover, muscle power is gaining consideration as an outcome measure of muscle function in COPD due to its strong association with mobility and function^8,11^. However, there is a lack of knowledge about the same muscle properties in COPD, whereas their change in response to a conventional pulmonary rehabilitation (PR) program may clarify the skeletal muscle dysfunction in COPD, thus improving the design and individualization of the exercise intervention.

Since changes in skeletal muscle function evaluated with both specific strength measurements and functional tests are still not well explored, the primary objective of the present study was to measure changes in different properties of skeletal muscles (i.e., strength, power, control, and activation) and functional performance after a conventional PR program and three months of follow-up in outpatients with COPD. Furthermore, the secondary objective of the study was to evaluate the relationship and contribution of the muscle properties examined in changes in functional performance.

## Methods

### Study design

The prospective observational trial conducted between January and October 2021 on outpatients with COPD attending supervised PR at a tertiary referral sub-acute rehabilitation centre. The study was approved in October 2020 by the Ethics Committee of the IRCCS Fondazione Don Carlo Gnocchi and was conducted in accordance with the Declaration of Helsinki. All participants signed an informed consent form before study participation. Trial registration: NCT04767126.

### Participants

Inclusion criteria were: (1) COPD diagnosis (Global Initiative for Chronic Obstructive Lung Disease (GOLD) stage: II-III-IV)^12^. (2) Male and female patients, 35–85 years old. Subjects were excluded from the study if they had respiratory diseases other than COPD, unstable clinical conditions, or exacerbations in the last four weeks. Patients with cardiologic, orthopaedic or neurological conditions limiting their ability to perform the exercise training or the assessments planned were also excluded from the study.

### Procedure

Participants attended 15 sessions of supervised PR. During the first session, medical history and baseline measurements were collected (T0). The assessment procedure, except the 6-min walk test (6MWT), was repeated at the conclusion of the PR program (T1) and after three months from the completion of the PR program (T2).

### Measurements

Bioimpedance analysis (RD-545-sv, Tanita, The Netherlands) was used to obtain the fat-free mass index (FFMI; i.e., fat-free mass/height^2^), where scores < 17 kg/m^2^ for men and < 15 kg/m^2^ for women were considered as low^13^. In addition, muscle quality of the dominant limbs was calculated^14^.

The total distance covered during the 6MWT was measured for each participant following a standardized procedure^15^. In addition, the 6-min walk distance (6MWD) was expressed also as a percentage of the predicted value^16^.

The lower extremity function was evaluated using the Short Physical Performance Battery (SPPB)^17^. Furthermore, the mean functional muscle power of the lower limbs was estimated using previously reported equation that included time to complete 5STS, subjects’ body mass and height, and the height of the seat (0.43 m) as variables^18^.

Handgrip strength (HGS) of the dominant arm was assessed with a digital hand dynamometer (Jamar Plus + Dynamometer, Performance Health Supply, USA) by three maximal contractions of 3 s each, separated by 60 s of rest^19^.

Quadriceps muscle function was measured with a computerized dynamometer (Biodex System 4 Pro, Biodex Medical Systems Inc, USA) in the dominant lower limb. First, the maximal isometric peak torque (PT) was recorded at 65° of knee flexion during a contraction of 4 s, repeated for 3 attempts with 60 s of rest. The percentage of predicted values for quadriceps PT was calculated using a previously reported equation^20^. Values < 70% of the predicted account for quadriceps muscle weakness^21^. Then, force steadiness was measured during sub-maximal isometric contractions of the dominant quadriceps, using visual feedback set at 30% of the patient's PT, during 5-s contractions^9^. Five familiarization trials were performed followed by five attempts, each one separated by 15 s of rest. Quadriceps PT and RFD were normalized to fat-free mass, and force steadiness was computed as the coefficient of variation of force by normalizing the standard deviation (SD) to the mean force.

Muscle activation and kinematic parameters were recorded during strength measurements and the SPPB test using a wireless system (Cometa Wave Plus, Cometa S.r.l., Italy) with surface electromyography (EMG) and inertial measurement units (IMUs)^22^. After standard skin preparation, EMG electrodes were placed on the rectus femoris of both legs, vastus lateralis and medialis of the dominant leg, and flexor carpi and biceps brachii of both arms. In addition, eight IMUs were attached via elastic bands to the thighs, shanks and feet of both lower limbs, as well as on the pelvis and chest of the patient. The EMG signals from each trial were full-wave rectified and converted into their root mean square (RMS) amplitude. Values of RMS were used to evaluate the maximal muscle activation during tests of quadriceps PT, HGS, and 5STS for both the concentric (i.e., sit-to-stand) and eccentric (i.e., stand-to-sit) phases, as well as to measure the submaximal muscle activation during the test of force steadiness. Results of maximal muscle activation obtained during isometric testing were then normalized to quadriceps PT values, whereas values of maximal muscle activation of 5STS and force steadiness were normalized to the maximum RMS recorded during isometric PT. This ratio (i.e., maximal muscle activation of 5STS/maximal muscle activation during PT) represents indexes of relative muscle activation during the concentric or eccentric phase of 5STS and neuromuscular economy, respectively^23,24^.

The overall dyspnea was measured with the Barthel index based on dyspnea and with the Medical Research Council (MRC) dyspnea scale^25^. The risk of death was also assessed with the Body-Mass, Airflow Obstruction, Dyspnea, and Exercise Capacity (BODE) index^26^. Further description of the assessment procedures and data processing is provided in the Supplementary Information.

### Pulmonary rehabilitation program

The conventional PR program consisted of 90 min sessions, 3 days per week, including both training with a cycle ergometer and resistance training with free weights or elastic bands. The workload for the cycle ergometer was calculated using the 6MWD to estimate the peak work rate according to a previously reported equation^27^. Then, exercise intensity for the first training session was set at 50% of the estimated peak work rate, and increased by 10 W during the following sessions according to the level of dyspnea and fatigue as described elsewhere^28,29^.

Resistance training started with the maximal load that could be lifted during 20 repetitions for 2 sets, and was weekly increased until achieving the maximal load that could be lifted for 10 repetitions and 3 sets.

Airway clearance techniques were adopted if necessary. Finally, participants received educational support for maintaining regular exercise and physical activities after the conclusion of the PR program. Further details of the PR program are available in the Supplementary Information.

### Statistical analysis

The sample size calculation was performed considering the mean functional muscle power estimated during the 5STS test as validated by Alcazar et al.^18^. Therefore, assuming an increase ≥ 25% in muscle power, with a 25% dropout rate, a sample size of 20 subjects was found (α = 0.05; power = 0.8).

Results were reported as mean and SD, unless otherwise stated. All variables were tested for the normality of the distribution. Variables assessed only pre- and post-PR program were analyzed with paired t-test for normally distributed data, and with Wilcoxon’s signed-rank test in case of not normally distributed data. According to Cohen, the estimated effect size (d) of the treatment was considered small (d = 0.2), moderate (d = 0.5), or large (d = 0.8)^30^.

The outcome measures assessed at all the three time points were analyzed using mixed-models analysis of variance (ANOVA). The models included time as a fixed effect variable, and participant or participant per time interaction as random effects variables. The Akaike information criterion was used to evaluate the fit of models with different covariance structures^31^. Post hoc tests compared each of the three time points by using Sidak adjustments to control for Type I error.

Relationships between pre- and post-PR (Δ) changes for variables of muscle function and functional performance were tested using Pearson's correlation coefficient (r). Values of r were considered moderate (0.3 > r < 0.5), large (0.5 > r < 0.7), and very large (0.7 > r < 0.9). Relationships for the same variables at each time point were also tested with linear mixed models. The models were set as for ANOVA analysis, but labelling one variable as dependent variable, and the other as covariate.

Based on significant univariate correlations, multiple linear regression analysis included Δ changes in quadriceps function (i.e., leg muscle quality, PT, RFD, and relative activation during 5STS) as independent variables, and Δ changes in functional performance (i.e., 6MWD, 4mGS, 5STS time and power) as dependent variables. Significant predictors from previous models were then used in a model with baseline independent variables of sex, age in years, and percentage predicted forced expiratory volume in 1 s (FEV_1_). The stepwise backward method was used to enter data into the models and the assumption of multicollinearity was checked. As general rule, the number of patients analyzed was 5 times the number of variables entered into the multiple regression equation. Statistical analyses were performed using SPSS Statistics software (IBM, Chicago, IL, USA).

## Results

Twenty outpatients, 13 men and 7 women, with mild to severe COPD and increased mean impedance of the respiratory system completed the study (Table 1). Two patients missed the follow-up visit for difficulties with transportation. At baseline, participants showed reduced functional exercise capacity and preserved maximal muscle strength of the lower limbs, although 6 out of 20 patients exhibited weakness of quadriceps muscle (Table 2). The mean FFMI score was also nearly normal, although five female patients showed low baseline values. None of the participants complained of any adverse event or discomfort during the assessments or the PR program.

**Table 1:** Demographic and clinical characteristics of the patients.

	*N* = 20
	Mean (SD)
Age, years	71 (6)
Females, n (%)	7 (35)
Pack-years smoked, n	46.80 (28.60)
FEV_1_, % predicted	52.40 (20.70)
FVC, % predicted	90.80 (22.45)
FEV_1_/FVC, %	45.39 (15.15)
R_TOT,_ cmH_2_O∙s∙L ^−1^	4.59 (1.97)
R_TOT,_ % predicted	145.71 (35.70)
X_TOT,_ cmH_2_O∙s∙L ^−1^	− 2.69 (1.84)
X_TOT,_ % predicted	113.82 (14.94)
GOLD stage 2, *n* (%)	6 (30)
GOLD stage 3, *n* (%)	8 (40)
GOLD stage 4, *n* (%)	6 (30)
CRP, mg/L	2.09 (1.39)
Albumin, g/L	43.00 (2.60)
NLR, × 10^3^/µL	2.26 (0.87)

*CRP* c-reactive protein, *FEV*_*1*_ forced expiratory volume in 1 s, *FVC* forced vital capacity, *GOLD* Global Initiative for Chronic Obstructive Lung Disease, *NLR* neutrophil–lymphocyte ratio, *R*_*TOT*_ mean respiratory system resistance, *X*_*TOT*_ mean respiratory system reactance.

**Table 2:** Baseline functional characteristics of the patients.

	*N* = 20
	Mean (SD)
**Functional exercise capacity**	
6MWD, m	361.40 (109.95)
SPPB score	11.45 (0.76)
**Body composition**	
FFMI, kg/m^2^	17.38 (2.94)
**Muscle function**	
HGS, kg	30.83 (7.51)
Quadriceps PT, Nm	104.05 (44.84)
Quadriceps PT, % predicted	88.27 (42.40)

*6MWD* 6-min walk distance, *FFMI* fat-free mass index, *HGS* handgrip strength, *PT* peak torque, *SPPB* Short Physical Performance Battery.

### Changes in outcome measures after PR

After the completion of the PR program, 6MWD improved by 8% with a large effect size (Table 3). Furthermore, the Barthel index based on dyspnea, the MRC dyspnea scale, and the BODE index significantly decreased of 24.5% (with a large effect size), 25.9%, and 24.7%, respectively.

**Table 3:** Changes in outcome measures assessed at baseline and at the end of PR program.

	Baseline	End of PR	Mean difference	*p*	*d*
	*N* = 20	*N* = 20			
6MWD, m^a^	361.40 (109.95)	390.20 (107.21)	28.80 (35.94)	0.002	0.80
BID^a^	15.10 (14.19)	11.40 (12.15)	− 3.70 (4.29)	0.001	− 0.86
MRC^b^	3.00 (2.00, 4.00)	2.00 (1.00, 4.00)	− 1.00 (− 1.00, 0)	< 0.001^c^	
BODE^b^	4.00 (1.00, 10.00)	3.00 (0, 8.00)	− 1.00 (− 2.00, 0)	< 0.001^c^	

^a^Results presented as mean and (SD); ^b^Results presented as median (minimum, maximum); ^c^Wilcoxon signed-rank test; *6MWD* 6-min walk distance, *BID* Barthel Index based on dyspnea, *BODE* the body mass index, degree of airflow obstruction, dyspnea, and Exercise Capacity Index, *MRC* Medical Research Council dyspnea scale, *PR* pulmonary rehabilitation; SE, standard error.

### Changes in outcome measures after PR and follow-up

**Figure 1:**
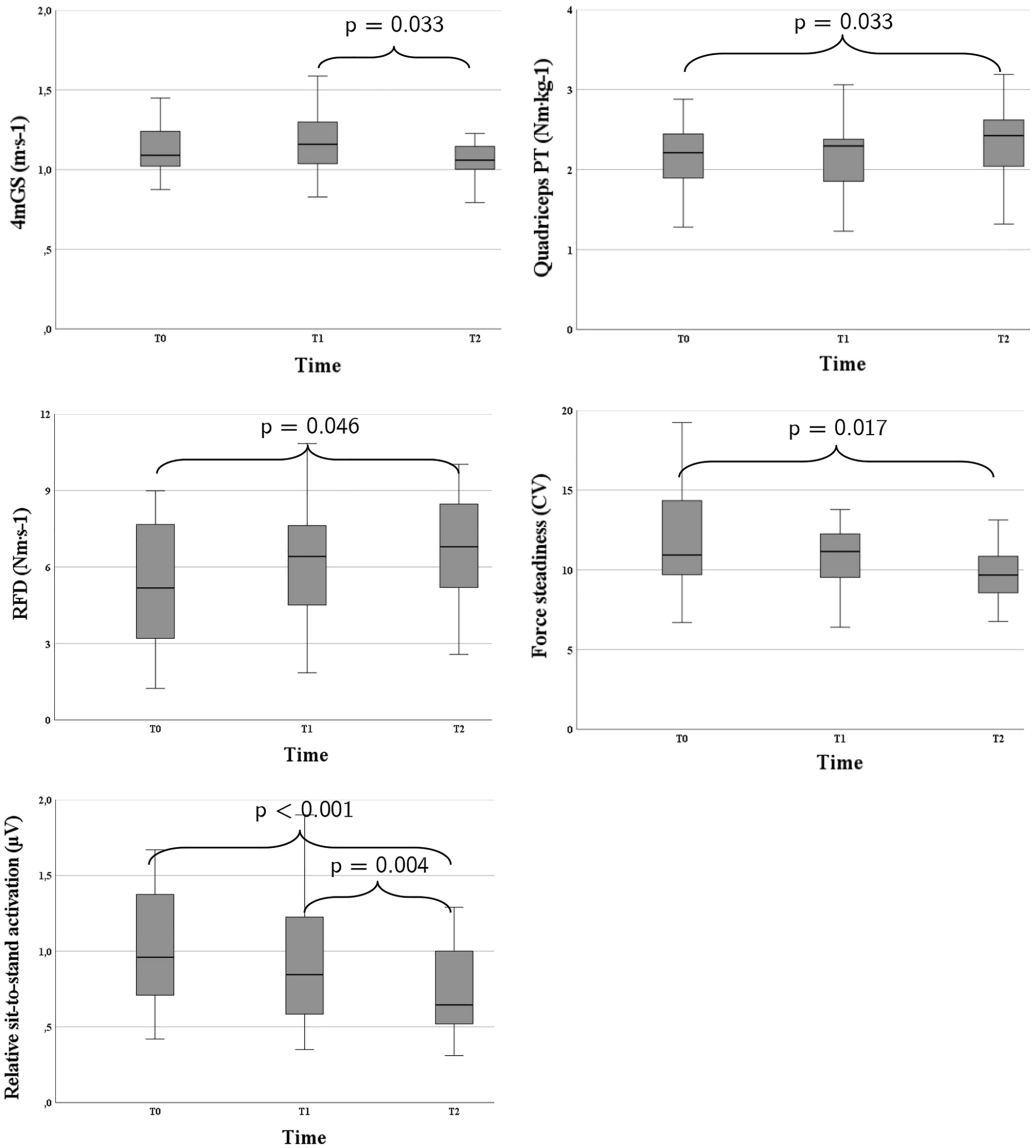
Boxplots of the significant differences in outcome measures at baseline (T0), at completion (T1) and 3 months post-completion of the PR program (T2). Dispersion bars represent standard deviations. *4mGS* 4-m gait speed test, *PT* peak torque, *RFD* rate of force development.

In the mixed-model ANOVA, a significant main effect of time was found in the 4mGS (F_2,37.093_ = 3.61, *p* = 0.037). Post-hoc pairwise comparisons identified a significant reduction of 7.7% in 4mGS between the end of PR and follow-up (Fig. 1). Time also showed a significant effect on quadriceps PT (F_2,37.937_ = 4.27, *p* = 0.021) and RFD (F_2,37.839_ = 3.464, *p* = 0.042). The pairwise comparison for both quadriceps PT and RFD showed a significant difference between T0 and T2 of 10.4% and 27.3%, respectively. There was a significant effect of time on force steadiness (F_2,37.220_ = 4.277, *p* = 0.021), with a significant decrease of 15.2% in the pairwise comparison between baseline and follow-up. The relative muscle activation during sit-to-stand also changed significantly over time (F_2,38.198_ = 9.689, *p* < 0.001), with a post-hoc significant reduction of 31.1% and 26% between T0-T2, and T1-T2, respectively. None of the other outcome measures showed significant effects of time. Descriptive ANOVA statistics are presented in the Supplementary Information (Table 1s).

### Relationships between outcome measures

**Figure 2:**
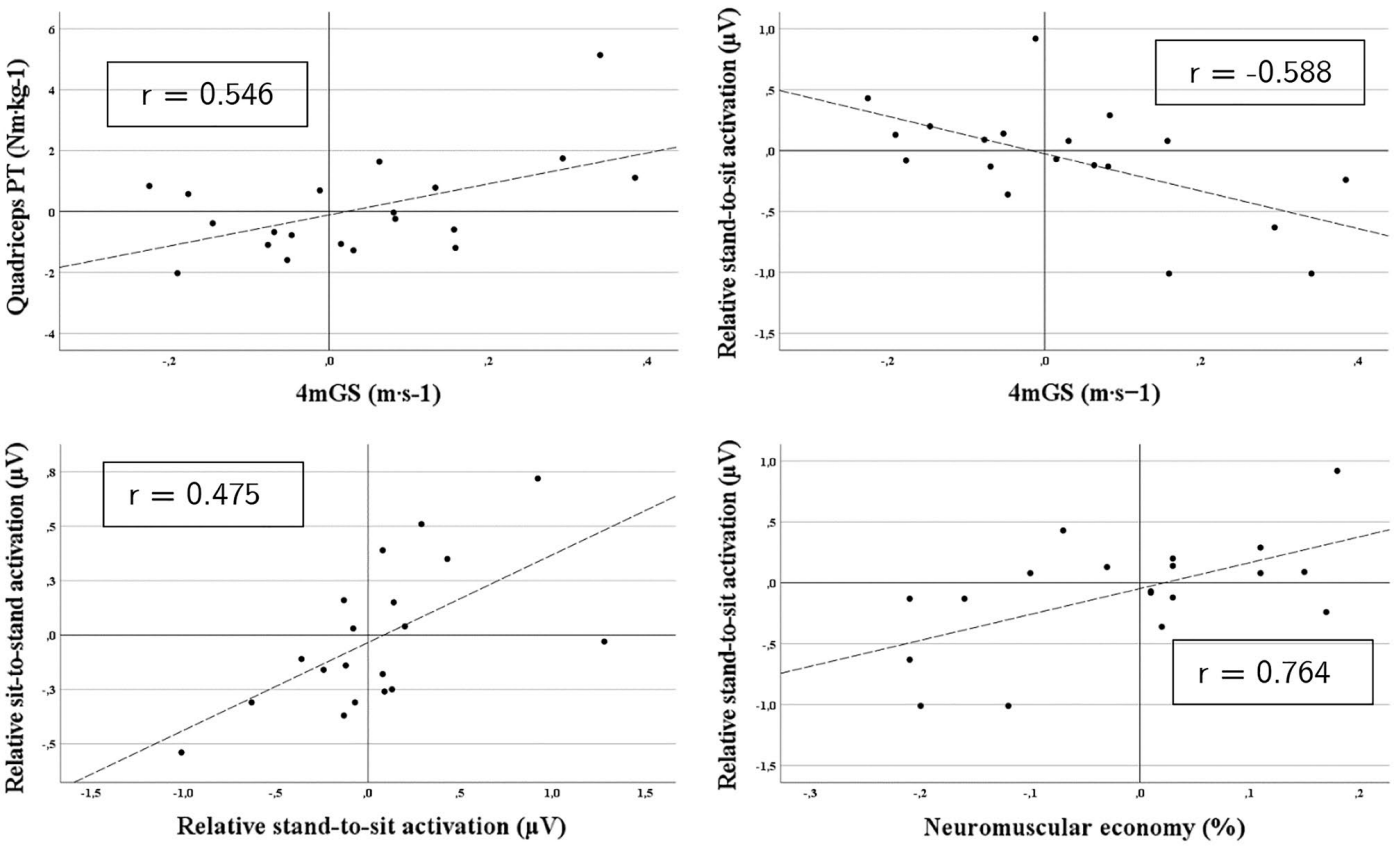
Pearson correlations between pre- and post-PR changes in outcome measures. *4mGS* 4-m gait speed test, *PT* peak torque, *RFD* rate of force development.

Pearson's correlations between Δ changes in 4mGS, quadriceps PT (*p* = 0.013), and relative stand-to-sit activation (*p* = 0.008) were both large (Fig. 2). In addition, there was a very large relationship between relative stand-to-sit activation and neuromuscular economy (*p* < 0.001), and a moderate correlation between relative stand-to-sit and sit-to-stand activation (*p* = 0.040).

Linear mixed models showed a relationship of magnitude (β) = 0.001 ms between 4mGS and leg muscle quality (*p* = 0.038). Therefore, a patient who improved by one unit his leg muscle quality, will have a predicted improvement in 4mGS by 0.001 ms, or two subjects whose leg muscle quality differed by one unit will have a predicted 4mGS difference of 0.001 ms. Similarly, 4mGS was related to quadriceps PT (β = 0.023 ms, *p* = 0.038) and RFD (β = 0.020 ms, *p* = 0.029). Conversely, there was a negative relationship between time to complete the 5STS test and leg muscle quality (β =  − 0.007 s, *p* = 0.010), quadriceps PT (β =  − 0.288 s, *p* = 0.009), and RFD (β =  − 0.359 s, *p* < 0.001). Furthermore, there was a positive relationship between mean functional power during 5STS and RFD (β = 8.084 W, *p* = 0.007).

Multiple linear regression showed that Δ changes in relative quadriceps activation during stand-to-sit can significantly predict Δ changes in 4mGS (F_2,15_ = 4.391, *p* = 0.033) with an adjusted R^2^ of 0.29. Participants predicted variation in 4mGS was therefore equal to 0.024 + (− 0.192 ∙ relative stand-to-sit quadriceps activation). In addition, Δ changes in relative quadriceps activation during stand-to-sit included in a model with baseline variables of sex, age, and percentage of predicted FEV_1_ significantly predict Δ 4mGS with an adjusted R^2^ of 0.29 (F_1,17_ = 8.967, *p* = 0.008). Therefore, the predicted Δ change in 4mGS was equal to 0.015 + (− 0.225 ∙ relative stand-to-sit quadriceps activation). None of the models used for the other dependent variables showed significant predictors of quadriceps function. Details of multilinear regression analysis are reported in the Supplementary Information (Tables 2s and 3s).

## Discussion

This study aimed to describe changes in skeletal muscle properties and functional performance after PR and three months of follow-up in outpatients with COPD. We observed an improvement in 6MWD after PR, whereas there was a decline in gait speed three months after the conclusion of the PR program. Quadriceps muscle properties such as PT, RFD, force steadiness, and relative sit-to-stand activation improved between baseline and follow-up. Furthermore, we found that after PR, gait speed changes were related to quadriceps maximal strength and relative activation during stand-to-sit movement. Similarly, longitudinal changes in properties of quadriceps muscle after PR and follow-up were related to performance in gait speed and sit-to-stand performance. Of the examined quadriceps muscle properties, relative quadriceps activation during stand-to-sit was the only muscle property that might predict gait speed, intended as post-PR change. In addition, this study highlighted that, when added to a model with baseline determinants of functional capacity, post-PR changes in relative muscle activation of the quadriceps during the eccentric (stand-to-sit) phase of 5STS is the only significant predictor of variations of gait speed in outpatients with COPD.

### Changes in skeletal muscles function and functional performance after PR

Maintaining the benefits of training in terms of functional performance after the completion of PR programs represents a challenging issue in people with COPD^32^. A tendency to lose exercise-induced gains in functional performance after a short follow-up with no maintenance was observed also in the present study. Indeed, on one side we observed expected improvements in functional exercise capacity and dyspnea after conventional PR, and on the other side, there was a reduction in gait speed after three months of follow-up, similar to that reported in a previous study^33^. Conversely, amongst the muscle properties examined, maximal strength improved after the conclusion of the PR program. Although unexpected, similar findings were also reported by Ahnfeldt-Mollerup et al.^34^, who failed to achieve significant improvements in maximal isometric strength of the quadriceps after nine weeks of conventional PR in patients with COPD, but observed an increasing trend in the same value after three months of follow-up.

The quadriceps explosive strength (i.e., RFD) showed a similar trend as quadriceps PT at each time point in the study. In adult populations RFD has been reported to be more sensitive to changes in neuromuscular function and better related to daily tasks than maximal muscle strength^35,36^. In the last few years, RFD received attention also in COPD-related research, and two previous studies reported improvements in this muscle property after specific resistance training^37,38^. Our findings also showed a not significant improvement in explosive strength after a conventional PR program. We hypothesized that the failure to improve both maximal strength and explosivity after PR may be attributed to the adoption of a non-specific resistance training protocol rather than using training regimens focused, for example, on heavy resistance or power of the lower limbs^38^. At the same time, we reported a preserved maximal strength for the majority of patients at baseline, whereas maximal strength has been shown to improve more in weaker individuals with COPD^39,40^. Moreover, the additional improvement in quadriceps PT and RFD at follow-up may have been related to the participation in daily living or physical activities of each patient, but this remains to be elucidated.

To the best of our knowledge, this is the first study evaluating changes in submaximal force control, acquired during a steadiness task, in individuals with COPD. Force steadiness (i.e., fluctuations of force signal) is expected to decrease following exercise training; nevertheless, previous studies on elderly adults reported contrasting results on the effects of both high- or low-intensity resistance training on force steadiness during submaximal isometric contractions^9,41,42^. In the present study, we also observed an unexpected improvement from baseline to follow-up in force steadiness, without significant changes after PR. However, the increased submaximal force control was not associated with an improvement in EMG values of the neuromuscular economy (i.e., a reduction in the normalized EMG signal at the same absolute load) after PR. Previous authors reported a greater reduction in force fluctuations in weaker subjects. Therefore, the higher proportion of patients with preserved quadriceps strength in the present study might have shown a reduced response to the training stimulus^41,42^.

Another novelty of this study is the evaluation of quadriceps muscle activation during the 5STS test^23^. We observed a substantial reduction between baseline and follow-up in the relative (normalized to maximal isometric activation of the quadriceps) muscle activation during the sit-to-stand phase in patients with COPD. Since higher values would indicate a greater muscle activation requirement, thus, patients were presumably able to perform the same task with less difficulty at the end of the PR program. The previously mentioned increase in the maximal isometric strength of the quadriceps may also have contributed to this result. Indeed, there are two possible explanations for the decrease of relative sit-to-stand activation: (1) an increase in the maximal isometric activation of the quadriceps with no change in muscle activation required for standing transition or, vice-versa, (2) a decrease in the quadriceps activation during sit-to-stand without any variation in maximal isometric activation of the quadriceps. As a matter of fact, we did not detect significant changes in the maximal isometric activation of the quadriceps at any time point of the study, therefore the second hypothesis may be more plausible.

### Role of skeletal muscle function in changes in functional performance after PR

Regarding the secondary objective of the study, we found that changes in usual gait speed after PR are positively related to changes in maximal isometric strength of the quadriceps. In this context, a recent review was unable to find a relationship between quadriceps strength and gait speed in COPD-related studies^5^. The present study, in contrast, highlighted the possible usefulness of measuring variations in walking speed to monitor improvements in muscle strength after PR in outpatients with COPD. In addition, we found a negative relationship between post-PR changes in gait speed and relative muscle activation during the eccentric phase (i.e., stand-to-sit) of 5STS, indicating that eccentric strength may contribute to functional performance, as previously reported in patients with COPD^42^. We cannot exclude, however, that force control may have affected the stand-to-sit performance more than the muscle strength of the lower limbs^44^.

Longitudinal relationships between outcomes at each time point of the study revealed an association between RFD and all the measures of functional performance. Interestingly, RFD but not quadriceps PT was related to functional muscle power, hence specific muscle power training during PR may require moderate-intensity but high-velocity of execution^45^.

Lastly, the present study aimed to determine the contribution of skeletal muscle properties to the improvement in functional performance after PR in persons with COPD. In this regard, we found that relative eccentric quadriceps activation was the only significant predictor of changes in gait speed, also when added to basic determinants^8,46^. Therefore, although there may be many more confounding factors than those considered in the present study our results may indicate a relevant contribution of eccentric force in determining an improvement in functional performance in patients with COPD^43^.

This study has several limitations. The limited number of patients that showed muscle weakness at baseline may have had greater improvements in various outcomes after PR^39^. Conversely, the majority of participants with preserved quadriceps strength may have produced a ceiling effect that limited significant improvements in muscle function measures after PR. Also, we cannot rule out that grading patients with the new ABCD methodology by GOLD^12^, might have better explained their preserved muscle strength and the possible ceiling effect on the results. For the same reason, discrepancies in levels of physical activity after the end of the PR program among these types of patients may have influenced follow-up results. Therefore, the generalizability of the results might be limited to those patients with preserved muscle strength. In addition, the length of the PR program adopted was shorter than the median length of PR programs in Europe^47^, and may have produced an insufficient stimulus to detect significant findings. However, the duration of the PR program was as long as allowed by the National Health Care System for outpatients with COPD. Besides, some technical limitations have to be addressed: first, we measured only isometric maximal strength, whereas dynamic strength might be better associated with functional performance^8^. However, the clinical setting where the study took place was time-limited and did not allow for multiple strength assessments. Second, the modality adopted to synchronize EMG and dynamometer signals may have caused variability within repeated measures and between patients. Finally, RFD was calculated only for the 200 ms time window, whereas shorter time intervals may have produced different results^35^.

## Conclusions

In this study, the benefits of conventional PR on functional performance appeared to decline after three months of follow-up in patients with COPD. The same did not seem to occur for various properties of the quadriceps muscle. These changes in functional performance and muscle properties appeared to be related; the eccentric quadriceps activation, in particular, might predict changes in functional performance. Therefore, PR should address resistance training by providing exercise protocols focused on different muscle properties, including eccentric contractions, in clinical COPD practice. Consequently, there is a need for further studies with interventional design focusing on eccentric resistance training compared with conventional resistance training in individuals with COPD.

## Supplementary Information

Supplementary Information.

## Data Availability

The data that support the findings of this study are available from the corresponding author upon reasonable request.

## References

[1] Maltais F (2014). An official American thoracic society/european respiratory society statement: Update on limb muscle dysfunction in chronic obstructive pulmonary disease. Am. J. Respir. Crit. Care Med..

[2] Singer J (2011). Respiratory and skeletal muscle strength in COPD: impact on exercise capacity and lower extremity function. J. Cardiopulm. Rehabil. Prev..

[3] Vestbo J (2006). Body mass, body mass, fat-free body mass, and prognosis in patients with chronic obstructive pulmonary disease from a random population sample. Am. J. Respir. Crit. Care Med..

[4] Seymour J (2010). The prevalence of quadriceps weakness in copd and the relationship with disease severity. Eur. Respir. J..

[5] Mathur S, Dechman G, Bui K-L, Camp PG, Saey D (2019). Evaluation of limb muscle strength and function in people with chronic obstructive pulmonary disease. Cardiopulm. Phys. Ther. J..

[6] Johnston KN, Potter AJ, Phillips A (2017). Measurement properties of short lower extremity functional exercise tests in people with chronic obstructive pulmonary disease: systematic review. Phys. Ther..

[7] Pancera S, Lopomo NF, Bianchi LNC, Pedersini P, Villafañe JH (2021). Isolated resistance training programs to improve peripheral muscle function in outpatients with chronic obstructive pulmonary diseases: A systematic review. Healthcare.

[8] Gephine S (2021). Specific contribution of quadriceps muscle strength, endurance, and power to functional exercise capacity in people with chronic obstructive pulmonary disease: a multicenter study. Phys. Ther..

[9] Hortobágyi T, Tunnel D, Moody J, Beam S, DeVita P (2001). Low- or high-intensity strength training partially restores impaired quadriceps force accuracy and steadiness in aged adults. J. Gerontol. Ser. A Biol. Sci. Med. Sci..

[10] Laidlaw DH, Bilodeau M, Enoka RM (2000). Steadiness is reduced and motor unit discharge is more variable in old adults. Muscle Nerve.

[11] Bui K-L (2019). Reliability of quadriceps muscle power and explosive force, and relationship to physical function in people with chronic obstructive pulmonary disease: an observational prospective multicenter study. Physiother. Theory Pract..

[12] Vogelmeier CF (2017). Global strategy for the diagnosis, management and prevention of chronic obstructive lung disease 2017 report: GOLD executive summary. Respirology.

[13] de Blasio F (2018). Malnutrition and sarcopenia assessment in patients with chronic obstructive pulmonary disease according to international diagnostic criteria, and evaluation of raw BIA variables. Respir. Med..

[14] Newman AB (2003). Strength and muscle quality in a well-functioning cohort of older adults: The health, aging and body composition study. J. Am. Geriatr. Soc..

[15] Enright PL (2003). The six-minute walk test. Respir. Care.

[16] Enright PL, Sherrill DL (1998). Reference equations for the six-minute walk in healthy adults. Am. J. Respir. Crit. Care Med..

[17] Medina-Mirapeix F (2016). Interobserver reliability of peripheral muscle strength tests and short physical performance battery in patients with chronic obstructive pulmonary disease: a prospective observational study. Arch. Phys. Med. Rehabil..

[18] Alcazar J (2018). The sit-to-stand muscle power test: an easy, inexpensive and portable procedure to assess muscle power in older people. Exp. Gerontol..

[19] Calik-Kutukcu E (2017). Arm strength training improves activities of daily living and occupational performance in patients with COPD. Clin. Respir. J..

[20] Decramer M, Gosselink R, Troosters T, Verschueren M, Evers G (1997). Muscle weakness is related to utilization of health care resources in COPD patients. Eur. Respir. J..

[21] Vaes AW (2021). The correlation between quadriceps muscle strength and endurance and exercise performance in patients with COPD. J. Appl. Physiol..

[22] Zucchelli A, Pancera S, Bianchi LNC, Marengoni A, Lopomo NF (2022). Technologies for the instrumental evaluation of physical function in persons affected by chronic obstructive pulmonary disease: a systematic review. Sensors.

[23] Petrella JK, Kim J, Tuggle SC, Hall SR, Bamman MM (2005). Age differences in knee extension power, contractile velocity, and fatigability. J. Appl. Physiol..

[24] Cadore EL (2013). Neuromuscular adaptations to concurrent training in the elderly: effects of intrasession exercise sequence. Age.

[25] Vitacca M (2016). Development of a Barthel Index based on dyspnea for patients with respiratory diseases. Int. J. Chron. Obstruct. Pulmon. Dis..

[26] Celli BR, Casanova C, Plata VP (2004). The body-mass index, airflow obstruction, dyspnea, and exercise capacity index in chronic obstructive pulmonary disease. N. Engl. J. Med..

[27] Luxton N, Alison JA, Wu J, Mackey MG (2008). Relationship between field walking tests and incremental cycle ergometry in COPD. Respirology.

[28] Maltais F (1997). Intensity of training and physiologic adaptation in patients with chronic obstructive pulmonary disease. Am. J. Respir. Crit. Care Med..

[29] Pancera S (2021). Effectiveness of continuous chest wall vibration with concurrent aerobic training on dyspnea and functional exercise capacity in patients with chronic obstructive pulmonary disease: a randomized controlled trial. Arch. Phys. Med. Rehabil..

[30] Cohen J (1992). Quantitative methods in psychology. Psychol Bull..

[31] Akaike H (1974). A new look at the statistical model identification. IEEE Trans. Autom. Control.

[32] Spencer LM, McKeough ZJ (2019). Maintaining the benefits following pulmonary rehabilitation: Achievable or not?. Respirology.

[33] Kon SSC (2014). The 4-metre gait speed in COPD: responsiveness and minimal clinically important difference. Eur. Respir. J..

[34] Ahnfeldt-Mollerup P (2015). The effect of protein supplementation on quality of life, physical function, and muscle strength in patients with chronic obstructive pulmonary disease. Eur. J. Phys. Rehabil. Med..

[35] Aagaard P, Simonsen EB, Andersen JL, Magnusson P, Dyhre-Poulsen P (2002). Increased rate of force development and neural drive of human skeletal muscle following resistance training. J. Appl. Physiol..

[36] Maffiuletti NA, Bizzini M, Widler K, Munzinger U (2010). Asymmetry in quadriceps rate of force development as a functional outcome measure in TKA. Clin. Orthop..

[37] Hoff J (2007). Maximal strength training of the legs in COPD: a therapy for mechanical inefficiency. Med. Sci. Sports Exerc..

[38] Alcazar J (2019). Effects of concurrent exercise training on muscle dysfunction and systemic oxidative stress in older people with COPD. Scand. J. Med. Sci. Sports.

[39] Garrod R, Marshall J, Barley E, Jones PW (2006). Predictors of success and failure in pulmonary rehabilitation. Eur. Respir. J..

[40] Jones SE (2015). Sarcopenia in COPD: prevalence, clinical correlates and response to pulmonary rehabilitation. Thorax.

[41] Tracy BL, Enoka RM (2006). Steadiness training with light loads in the knee extensors of elderly adults. Med. Sci. Sports Exerc..

[42] Kobayashi H, Koyama Y, Enoka RM, Suzuki S (2014). A unique form of light-load training improves steadiness and performance on some functional tasks in older adults. Scand. J. Med. Sci. Sports.

[43] Butcher SJ (2012). Associations between isokinetic muscle strength, high-level functional performance, and physiological parameters in patients with chronic obstructive pulmonary disease. Int. J. Chron. Obstruct. Pulmon. Dis..

[44] Janssens L (2014). Impaired postural control reduces sit-to-stand-to-sit performance in individuals with chronic obstructive pulmonary disease. PLoS ONE.

[45] American College of Sports Medicine position stand (2009). Progression models in resistance training for healthy adults. Med. Sci. Sports Exerc..

[46] Rausch-Osthoff A-K, Kohler M, Sievi NA, Clarenbach CF, van Gestel AJ (2014). Association between peripheral muscle strength, exercise performance, and physical activity in daily life in patients with chronic obstructive pulmonary disease. Multidiscip. Respir. Med..

[47] Spruit MA (2014). Differences in content and organisational aspects of pulmonary rehabilitation programmes. Eur. Respir. J..

