# Supplementary material for: Muscle function and functional performance after pulmonary rehabilitation in patients with chronic obstructive pulmonary disease: a prospective observational study
Source: Sci Rep. 2022 Sep 30;12:16386. doi: 10.1038/s41598-022-20746-y (PMC9525595; doi:10.1038/s41598-022-20746-y)
Supplement: Supplementary file 1 — Supplementary Information. [file 41598_2022_20746_MOESM1_ESM.pdf]

# Muscle function and functional performance after pulmonary rehabilitation in patients with chronic obstructive pulmonary disease: a prospective observational study

Simone Pancera[1*], Luca N. C. Bianchi[1], Roberto Porta[1], Jorge H. Villafañe[1], Riccardo Buraschi[1] and Nicola F. Lopomo[2]

[1]IRCCS Fondazione Don Carlo Gnocchi, Milan, Italy.

[2]Department of Information Engineering, University of Brescia, Brescia, Italy.

[*]Corresponding author: Simone Pancera, IRCCS Fondazione Don Carlo Gnocchi, via Capecelatro 66, 20148, Milan, Italy. Mail: s.pancera002@unibs.it.

Supplementary material. Muscle function and functional performance after pulmonary rehabilitation in patients with chronic obstructive pulmonary disease: a prospective observational study

## Measurements

### *Segmental body composition*

Participants stood barefoot on the scale holding its handles for as long as needed for the analysis. The device used equations not supplied by the manufacturer to calculate both total (i.e., whole-body) and segmental (i.e., for each extremity and trunk) percentages of fat, muscle mass, and bone mass (only whole-body) from the bioimpedance analysis readings.

### *Functional performance*

Six-minute walk test: after receiving standardized instructions, patients were asked to walk on an indoor 20-m flat corridor with the goal of covering the longest distance possible[1]. Oxygen saturation and heart rate were recorded during the test via a bluetooth oximeter (BlueNight Trainer, Sleepinnov Technology, France).

Short Physical Performance Battery (SPPB): includes three tests: 1) 5-repetitions STS (5STS), 2) standing balance, and 3) 4-meter gait speed (4mGS). The SPPB provides a score between zero and twelve points, with higher scores representing better performance. For 5STS participants were instructed to stand up from a standardized armless chair (measuring 0.43 m from ground to seat) as quickly as they can for five times, without stopping in between, and with arms crossed over the chest. The fastest of three attempts

separated by 1 minute of rest was recorded for analysis. For the balance test, subjects were asked to maintain 3 different standing positions for 10 seconds: first with the feet side-by-side, then in semi-tandem and finally in tandem. To perform 4mGS test, participants were instructed to walk along a four-meter course at their usual speed, and total walking time was recorded.

*Peripheral muscle strength*

Handgrip strength: during the testing procedure participant maintained the arm along the side, the elbow at 90 degrees of flexion and a neutral forearm position[2].

Quadriceps muscle function: after 3 minutes of warm-up on a stationary bicycle, the patient was positioned on the dynamometer seat with the axis of rotation of the dominant knee joint aligned to the axis of rotation of the dynamometer arm. The leg was secured in the cuff just 2 cm above the medial malleolus and the dynamometer lever arm was moved to 65° of knee flexion. 1) To measure isometric quadriceps peak torque (PT) participants were instructed to contract the quadriceps muscle as fast and strong as possible and received standardized encouragement during the attempt. 2) To assess force steadiness, patients were instructed to watch at a purple target line on the dynamometer monitor corresponding to 30% of their quadriceps PT. Subjects were then instructed to reach the target as fast as possible and to maintain the force as precisely as possible on the target during each contraction.

*Motion analysis*

Surface electromyography (EMG) were recorded by placing two round electrodes in parallel with the direction of the muscle fibers between the distal tendon and the innervation zone

of each muscle (Figure 1s, A). Each pair of electrodes was connected to a wireless receiver also attached to the skin. Also, Inertial measurement units (IMUs) were used to obtain kinematic parameters during testing procedure (Figure 1s, B). Before starting the measurement, IMUs were calibrated and the sensors axes were aligned with anatomical directions. Signals from EMG and the inertial sensors were synchronously acquired with a receiver unit at a sampling rate of 142 Hz. Real time EMG and IMUs data, joint angles estimation, and 3-dimensional body representation were displayed on a computer through the manufacturer's software (EMG and Motion Tools, Cometa S.r.l., Italy).

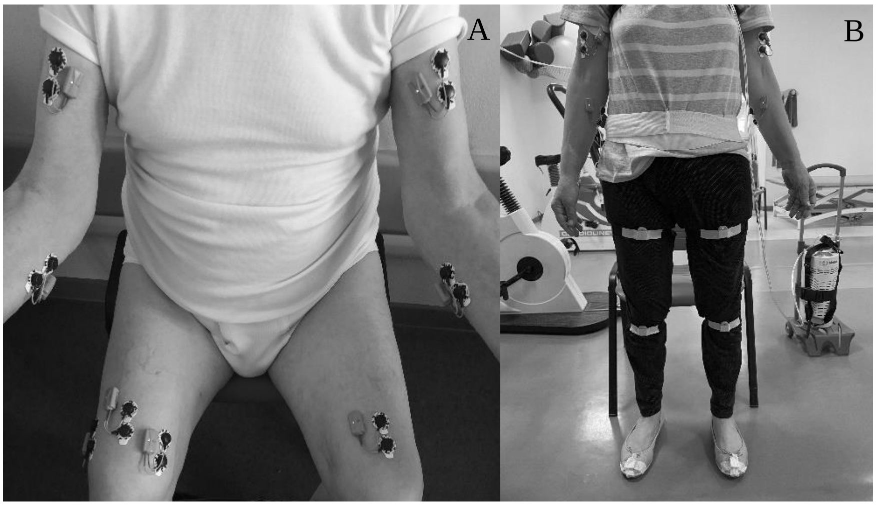


Figure 1s. Position of the EMG electrodes (A) and inertial sensors (B) on the patients.

Data processing

Leg muscle quality of the dominant lower extremity was calculated by dividing the PT of the dominant quadriceps muscle by the fat-free mass of the ipsilateral leg. Similarly, the arm muscle quality of the dominant upper extremity was obtained from the ratio of handgrip strength and the fat-free mass of the ipsilateral arm[3].

Data exported from the isokinetic dynamometer were plotted on a Microsoft Excel custom-made template in order to calculate the onset of contraction, rate of force

development (RFD), and target values for force steadiness. The onset of muscle contraction was defined as the time point at which the moment value exceeded the baseline torque by 2.5% of the maximum torque[4]. Then, RFD was derived from the torque-time curve recorded during quadriceps PT, and was calculated as the ratio of moment to time from the onset of muscle contraction up to 200 ms[5]. Finally, force steadiness was computed as the standard deviation of force fluctuation around the mean force, and then averaged within one trial[6]. Force steadiness analysis was done over five seconds of duration from the onset of muscle contraction and then averaged for the five trials and used in the statistical analysis.

During motion analysis processing, EMG and force signals were visually synchronized using the first spike of the acceleration signal from one IMU placed on the lever arm of the dynamometer. The beginning of the rise of the IMU signal was considered as the time point at which the quadriceps started to contract. The EMG signal was high-pass and low-pass filtered using a fourth-order Butterworth filter with cutoff frequencies of 6 and 400 Hz, respectively. Then, values of EMG were calculated for each muscle group involved in the single testing procedure and averaged within subjects. The maximal activation of the quadriceps was obtained from EMG parameters determined across a 100-ms window during the isometric test, considering the repetition with the higher PT value. Similarly, relative values of muscle activation were computed during both the concentric and eccentric phases of the fastest repetition of the 5-repetition sit-to-stand test, using a period of 100-ms centered on the specific knee angle (65°) at which quadriceps PT was assessed[7]. Values from the force steadiness task were determined for a 1-second period in the middle of the isometric contraction during the repetition with the lower value of steadiness. Finally, muscle activation obtained during the handgrip strength test was calculated from the

repetition with the highest force values over a 1-second period in the middle of the 3-seconds contraction.

Table 1s. Changes in outcome measures assessed after pulmonary rehabilitation and three months of follow-up.

| | Baseline | End of PR | Follow-up | Total | CV |
|---|---|---|---|---|---|
| | N = 20 | N = 20 | N = 18 | N = 58 | (%) |
| Functional performance | | | | | |
| 4mGS, $m \cdot s^{-1}$ | 1,14 (0,16) | 1,17 (0,20) | 1,08 (0,16) † | 1,13 (0,17) | 13.0 |
| 5STS, s | 9.58 (2.23) | 9.03 (1.36) | 9.45 (1.78) | 9.35 (1.82) | 19.4 |
| Functional power, W | 286.77 (111.94) | 288.13 (107.01) | 293.75 (105.94) | 289.41 (106.54) | 36.8 |
| Body composition | | | | | |
| FFMI, $kg/m^2$ | 17.38 (2.94) | 17.32 (2.91) | 17.82 (2.52) | 17.49 (2.77) | 15.8 |
| Leg muscle quality, $Nm \cdot kg^{-1}$ | 12.33 (4.54) | 12.67 (3.99) | 13.86 (3.12) | 12.92 (3.94) | 30.5 |
| Arm muscle quality, kg | 12.02 (1.86) | 12.94 (2.71) | 12.70 (2.33) | 12.56 (2.33) | 18.5 |
| Muscle contraction | | | | | |
| HGS, kg | 30.83 (7.51) | 31.81 (7.57) | 32.54 (7.17) | 31.70 (7.33) | 23.1 |

| | | | | | |
|---|---|---|---|---|---|
| Quadriceps PT, Nm·kg-1 | 2.03 (0.71) | 2.08 (0.61) | 2.33 (0.54) | 2.14 (0.63)* | 29.3 |
| RFD, Nm·s-1 | 5.31 (2.47) | 6.28 (2.32) | 6.76 (2.11) | 6.09 (2.35)* | 38.6 |
| Force steadiness, CV | 11.79 (3.17) | 11.00 (2.76) | 10.00 (2.27) | 10.96 (2.82)* | 25.7 |
| Muscle activation | | | | | |
| HGS maximal activation, µV·kg-1 | 6.60 (2.80) | 6.51 (3.14) | 6.53 (2.53) | 6.55 (2.80) | 42.7 |
| Quadriceps maximal activation, µV·Nm-1 | 1.46 (0.35) | 1.43 (0.23) | 1.48 (0.27) | 1.46 (0.28) | 19.4 |
| Neuromuscular economy, % | 35.68 (12.01) | 33.64 (10.22) | 28.99 (6.70) | 32.96 (10.24) | 31.1 |
| Maximal sit-to-stand activation, µV | 205.07 (87.70) | 198.39 (83.94) | 170.56 (64.99) | 191.71 (79.70)* | 41.6 |
| Maximal stand-to-sit activation, µV | 220.32 (74.36) | 237.36 (89.74) | 234.67 (71.05) | 230.65 (78.05) | 33.8 |

| | | | | | |
|---|---|---|---|---|---|
| Relative sit-to-stand activation, μV | 1.03 (0.38) | 0.96 (0.46) | 0.71 (0.28) † | 0.91 (0.40)* | 44.5 |
| Relative stand-to-sit activation, μV | 1.22 (0.48) | 1.12 (0.39) | 0.99 (0.39) | 1.11 (0.43) | 38.3 |

Results are presented as mean and (SD). * Significant difference between baseline and follow up ($p < 0.05$); † Significant difference between end of pulmonary rehabilitation program and follow up ($p < 0.05$); 4mGS, 4-meter gait speed test; 5STS, 5-repetition sit-to-stand test; CV, coefficient of variation; FFMI, fat-free mass index; HGS, handgrip strength; PT, peak torque; RFD, rate of force development.

Table 2s. Predictors of post-PR changes in 4mGS using muscle function parameters in multilinear regression analysis models

| | | B | SE | β | T | P | 95% CI | |
|---|---|---|---|---|---|---|---|---|
| 1 | Model | 0.01 | 0.03 | | 0.40 | 0.696 | -0.06 | 0.09 |
| | Leg muscle quality, $Nm \cdot kg^{-1}$ | -0.01 | 0.00 | -1.27 | -1.48 | 0.164 | -0.01 | 0.00 |
| | Quadriceps PT, $Nm \cdot kg^{-1}$ | 0.23 | 0.13 | 1.58 | 1.83 | 0.090 | -0.04 | 0.51 |
| | RFD, $Nm \cdot s^{-1}$ | 0.01 | 0.01 | 0.19 | 0.90 | 0.384 | -0.02 | 0.04 |
| | Relative quadriceps activation (stand-to-sit), μV | -0.19 | 0.09 | -0.48 | -2.21 | 0.046 | -0.38 | -0.00 |

| | | | | | | | | |
|---|---|---|---|---|---|---|---|---|
| 2 | Model | 0.02 | 0.03 | | 0.74 | 0.471 | -0.05 | 0.09 |
| | Leg muscle quality, $Nm \cdot kg^{-1}$ | -0.01 | 0.00 | -1.38 | -1.63 | 0.125 | -0.01 | 0.00 |
| | Quadriceps PT, $Nm \cdot kg^{-1}$ | 0.25 | 0.12 | 1.72 | 2.03 | 0.062 | -0.02 | 0.52 |
| | Relative quadriceps activation (stand-to-sit), µV | -0.16 | 0.08 | -0.41 | -2.03 | 0.062 | -0.33 | 0.01 |
| 3 | Model | 0.02 | 0.03 | | 0.71 | 0.490 | -0.05 | 0.10 |
| | Quadriceps PT, $Nm \cdot kg^{-1}$ | 0.06 | 0.03 | 0.37 | 1.82 | 0.089 | -0.01 | 0.12 |
| | Relative quadriceps activation (stand-to-sit), µV | -0.19 | 0.08 | -0.48 | -2.35 | 0.033 | -0.37 | -0.02 |

CI, confidence interval; PT, peak torque; RFD, rate of force development; SE, standard error. Significant predictors from the most suitable model are reported in bold characters.

Table 3s. Predictors of post-PR changes in 4mGS using baseline clinical parameters and post-PR changes of stand-to-sit relative quadriceps activation in multilinear regression analysis models

| | | B | SE | β | T | p | 95% CI | |
|---|---|---|---|---|---|---|---|---|
| 1 | Model | 0.11 | 0.50 | | 0.22 | 0.827 | -0.95 | 1.18 |
| | Age | -0.00 | 0.01 | -0.08 | -0.34 | 0.740 | -0.02 | 0.01 |
| | Sex | 0.07 | 0.08 | 0.19 | 0.87 | 0.402 | -0.10 | 0.24 |

| | | | | | | | | |
|---|---|---|---|---|---|---|---|---|
| | FEV$_1$ (% predicted) | 0.00 | 0.00 | -0.04 | -0.17 | 0.867 | -0.01 | 0.00 |
| | Relative quadriceps activation (stand-to-sit), μV | -0.21 | 0.08 | -0.56 | -2.59 | 0.022 | -0.39 | -0.04 |
| 2 | Model | 0.12 | 0.48 | | 0.25 | 0.810 | -0.90 | 1.14 |
| | Age | 0.00 | 0.01 | -0.09 | -0.44 | 0.664 | -0.02 | 0.01 |
| | Sex | 0.07 | 0.08 | 0.20 | 0.97 | 0.348 | -0.09 | 0.23 |
| | Relative quadriceps activation (stand-to-sit), μV | -0.21 | 0.08 | -0.56 | -2.70 | 0.016 | -0.38 | -0.05 |
| 3 | Model | -0.09 | 0.10 | | -0.90 | 0.383 | -0.30 | 0.12 |
| | Sex | 0.08 | 0.07 | 0.22 | 1.12 | 0.281 | -0.07 | 0.23 |
| | Relative quadriceps activation (stand-to-sit), μV | -0.22 | 0.08 | -0.58 | -2.99 | 0.009 | -0.38 | -0.07 |
| 4 | Model | 0.02 | 0.03 | | 0.45 | 0.659 | -0.06 | 0.09 |
| | Relative quadriceps activation (stand-to-sit), μV | -0.23 | 0.08 | -0.59 | -2.99 | 0.008 | -0.38 | -0.07 |

CI, confidence interval; FEV$_1$; forced expiratory volume in 1 second; SE, standard error. Significant predictors from the most suitable model are reported in bold characters.

## References

1. Holland, A.E. *et al*. An official European Respiratory Society/American Thoracic Society technical standard: field walking tests in chronic respiratory disease. *Eur. Respir. J*. 44, 1428–1446 (2014).

2. Calik-Kutukcu, E., *et al*. Arm strength training improves activities of daily living and occupational performance in patients with COPD. *Clin. Respir. J*. 11, 820–832 (2017).

3. Newman, A.B. *et al*. Strength and muscle quality in a well-functioning cohort of older adults: the health, aging and body composition study. *J. Am. Geriatr. Soc*. 51, 323–330 (2003).

4. Aagaard, P. *et al*. Increased rate of force development and neural drive of human skeletal muscle following resistance training. *J. Appl. Physiol*. 93, 1318–1326 (2002).

5. Maffiuletti, N.A. *et al*. Rate of force development: physiological and methodological considerations. *Eur. J. Appl. Physiol*. 116, 1091–1116 (2016).

6. Hortobágyi, T. *et al*. Low- or high-intensity strength training partially restores impaired quadriceps force accuracy and steadiness in aged adults. *J. Gerontol. Ser. A Biol. Sci. Med. Sci*. 56, B38–B47 (2001).

7. Petrella, J.K. *et al*. Age differences in knee extension power, contractile velocity, and fatigability. *J. Appl. Physiol*. 98, 211–220 (2005).
